# Current use and future potential of oscillometry in UK lung function testing: a national survey

**DOI:** 10.1136/bmjresp-2025-003786

**Published:** 2026-06-25

**Authors:** Madison E Geeves, Michael J Hughes, Harry S Griffin, Karl P Sylvester, Zoe L Saynor

**Affiliations:** 1 Respiratory and Sleep Physiology, Hampshire Hospitals NHS Foundation Trust, Winchester, UK; 2 Health Sciences, University of Southampton, Southampton, UK; 3 NIHR Southampton Biomedical Research Centre, Southampton, UK; 4 Respiratory Physiology, Papworth Hospital NHS Foundation Trust, Cambridge, UK; 5 Lung Function Unit, Cambridge University Hospitals NHS Foundation Trust, Cambridge, UK

**Keywords:** Surveys and Questionnaires, Respiratory Function Test, Asthma, Pulmonary Disease, Chronic Obstructive, Respiratory Measurement

## Abstract

**Background:**

Despite growing international momentum, uptake of oscillometry in routine lung function testing services in the United Kingdom (UK) is unclear.

**Objectives:**

Evaluate the current use of oscillometry in UK lung function services, identify key clinical applications, and explore barriers to wider adoption.

**Methods:**

A cross-sectional e-survey, including multiple-choice and free-text responses, was distributed to members of the Association for Respiratory Technology and Physiology (ARTP) and reported in line with the Checklist for Reporting Results of Internet E-Surveys (CHERRIES).

**Results:**

A total of 42 National Health Service (NHS) respiratory services responded. Of these, 40% reported owning an oscillometry device, equally split between forced oscillation technique (Resmon Pro) and impulse oscillometry (Vyntus IOS), with one using both. Oscillometry is already used in clinical practice in 69% of these services, most commonly for adults (57%) but also in paediatrics (21%) and both (21%). It is primarily used in asthma (100%) and chronic obstructive pulmonary disease (57%), and in 86% of cases for patients unable to perform technically acceptable spirometry. Among services without a device, 50% were actively considering purchasing within five years. The main barrier cited was funding (67%). A lack of clinical understanding was identified, with 86% of services perceiving respiratory consultants to have ‘little’ understanding of oscillometry, and 10% reporting ‘none’. Almost all respondents (95%) supported the development of national guidance from ARTP outlining benefits, limitations and reporting standards.

**Conclusion:**

Despite increasing interest, oscillometry is not yet routinely adopted across UK lung function services. It is predominantly used as an alternative to spirometry when technically acceptable results cannot be achieved and as an additional tool to support asthma care. This national survey highlights the need to improve awareness, training and standardisation. Collaboration with the ARTP to establish national guidance represents an important next step toward wider implementation in clinical practice.

**WHAT IS ALREADY KNOWN ON THIS TOPIC**

**WHAT THIS STUDY ADDS**

- Our data show that 40% of the surveyed National Health Service respiratory physiology services reported owning an oscillometry device, of which 69% actively use it within clinical practice, primarily for asthma and patients unable to perform spirometry.
- However, practice is inconsistent, with variability in test procedures, interpretation and confidence among healthcare professionals.

**HOW THIS STUDY MIGHT AFFECT RESEARCH, PRACTICE OR POLICY**

- There is a clear need to improve awareness, training and standardisation, and our findings strongly support the development of nationally endorsed guidance, through ARTP, to facilitate broader clinical adoption.

## Introduction

Chronic respiratory diseases, including asthma and chronic obstructive pulmonary disease (COPD), affect one in five people and are the third leading cause of death in the UK.^1^ Spirometry remains the ‘gold standard’ for diagnosing, monitoring and classifying the severity of these diseases.^2^ However, it requires significant patient effort and coordination which can limit feasibility in children, older adults and individuals with severe airflow limitation, motor or cognitive impairments.^3 4^ Moreover, spirometry is relatively insensitive to early or small airways disease, as forced expiratory volume in one second (FEV₁) predominantly reflects large airway function.^4^ This is particularly relevant in the small airways (≤ 2 mm diameter), which are often the earliest site of disease and may be affected before symptoms or spirometric abnormalities appear—a ‘silent zone’ of disease.^3 5^

Over the last several decades, alternative techniques have emerged to fill this diagnostic gap. These include multiple breath nitrogen washout (MBNW), specific airways resistance (sRaw) using whole-body plethysmography, hyperpolarised xenon MRI (Xe-MRI) and oscillometry, all of which assess lung function during tidal breathing and offer potential advantages in detecting early disease or in patients unable to complete spirometry.^6^,^8^ However, many of these techniques are limited by the need for medical gases, specialised and often bulky equipment, and are associated with high costs, thus making oscillometry more attractive.

Oscillometry, encompassing both the forced oscillation technique (FOT) and impulse oscillometry system (IOS), is a non-invasive method whereby sinusoidal or pulse sound waves are delivered at frequencies exceeding the normal breathing rate, to stimulate the entire respiratory system.^9^,^11^ It is a technique used to assess the mechanical properties of the upper and intrathoracic airways, lung parenchyma and chest wall during normal tidal breathing.^12^ It provides measures of airflow resistance (Rrs) and lung reactance (Xrs), reflecting airway calibre and the elasticity and inertial properties of the lung, respectively.^13^ Subsequently, the narrower the airways the higher the Rrs, and the greater the ‘stiffness’ the ‘more negative’ the Xrs.^3 12^ The range of frequencies covered by modern oscillometry devices allows for an independent assessment of both central and peripheral airways.^3^ While the higher frequencies (ie, 19 Hz and 20 Hz) are limited to the central airways, the lower frequencies (ie, 5 Hz) can penetrate deeper into the peripheral airways. Oscillometry has therefore been shown to identify the localisation of airflow obstruction,^3^ detect small airways dysfunction,^14^ assess within-breath inspiratory and expiratory parameters and thus detect expiratory flow limitation^15^ and provide a more sensitive assessment of bronchodilator response than spirometry.^16^

Although first described in the 1950s, recent technological advances and publication of international guidance have led to a resurgence of interest in oscillometry as a clinically viable test.^11 17^ Its ease of use, reduced time commitment and minimal requirement for patient effort and cooperation^18 19^ make it particularly relevant in individuals unable to perform forced manoeuvres.^11 20^ Moreover, its potential to identify early or subtle changes in the airways^3^ provides added value for early COPD and asthma investigations, especially when spirometry results are normal.^21^

Despite growing international momentum, uptake of oscillometry in routine UK respiratory practice remains unclear. The lack of established reference ranges and complexity regarding interpretation of results have been reported as probable barriers to its wider use^6^ and the European Respiratory Society (ERS) has recently called for greater standardisation.^12^ Additionally, UK respiratory stakeholders such as Asthma+Lung UK have highlighted oscillometry as a promising but under-used test.^22^

The aim of this study was to investigate the current use of oscillometry within UK respiratory services, including indications, equipment availability, perceived barriers and future intentions for adoption. This national survey was conducted in collaboration with the Association for Respiratory Technology and Physiology (ARTP) and is intended to act as a first step in informing future efforts toward guidance development, professional education and implementation.

## Methods

### Study design

This was a cross-sectional, online survey with an embedded mixed-methods design. Ethical approval was obtained from the University of Portsmouth Faculty of Science and Health Ethics Committee. The survey followed the Checklist for Reporting Results of Internet E-Surveys (CHERRIES)^23^ and the Checklist for Reporting Of Survey Studies (CROSS).^24^

### Patient and public involvement

Patients and/or the public were not involved in the design, conduct, reporting or dissemination of this study.

### Participant recruitment

The target population included healthcare professionals currently working in respiratory physiology departments in the UK and who are performing or overseeing lung function testing. This included registered or trainee respiratory physiologists, practitioner training programme (PTP) and/or scientist training programme (STP) students, clinical scientists and research practitioners or nurses.

The open survey link was initially disseminated electronically via email to all members of the ARTP and convenience sampling was used. To maximise reach, the survey link and accompanying advertisement (online supplemental material 1) were also shared through professional networks including LinkedIn, X (formerly Twitter), the ARTP website and forum, utilising a snowball sampling approach. Respondents were asked to ensure only one submission per respiratory physiology service or the National Health Service (NHS) trust.

All participants provided informed consent via a series of checkboxes after reading the study information. Participation was voluntary and responses were anonymous; however, participants were asked to identify their NHS Trust and, where applicable, hospital site to allow for the removal of potential duplicates. Completion and submission of the survey also constituted implied consent, as participants were informed that responses could not be withdrawn once submitted. No incentives were offered.

### Survey administration

The 30-item survey was developed by respiratory physiologists, clinical scientists and clinical academics and informed by international guidance documents,^12^ previously published literature^11 20^ and clinical experience. The survey captured key aspects of service delivery and practice, structured into four main sections: (1) service characteristics; (2) oscillometry use, indications for testing and barriers to adoption; (3) technical and interpretation considerations; and (4) training and education. It was piloted in house by the clinical team for clarity, usability and functionality and revised based on feedback. The survey was distributed in December 2023 with a follow-up reminder 2 weeks later, and remained open for one calendar month, closing in January 2024. It was administered in English using an online platform (onlinesurveys.ac.uk, JISC, Bristol, UK) and consisted of single-choice, multiple-choice questions (including checkboxes) and open-ended free-text responses. Adaptive questioning was used based on responses to previous items and mandatory items were highlighted before allowing respondents to proceed to the next section or final submission. Respondents were able to modify their answers prior to submitting the survey. A full copy of the questionnaire is provided in the online supplemental material (online supplemental material 2).

### Data analysis

Complete response data were exported into Microsoft Excel for analysis. Quantitative data were analysed using descriptive statistics and are presented as frequencies and percentages. Free-text responses were reviewed and grouped into broad thematic categories based on recurring content. No statistical correction methods were applied.

## Results

### Survey response and service characteristics

A total of 46 responses were received, with two international and two duplicate entries excluded, resulting in 42 valid UK-based responses. Responding services were all part of the NHS and located across England (83%), Scotland (10%), Wales (5%) and Northern Ireland (2%). The UK region and county were inferred from the reported NHS trust (table 1). Most services were based in secondary care (69%), followed by tertiary/specialist centres (29%) and one reportedly from primary care (2%). The majority were adult services (69%), with 12% paediatric and 19% mixed. The median number of full-time equivalent respiratory physiology staff in each service was 7.4 (range: 1–23).

**Table 1:** Geographical location for oscillometry device owners versus non-device owners

UK region	County	% (n) of device owners (n=17)	% (n) of non-device owners (n=24)
**England**			
London	Greater London	18 (4)	17 (4)
South East England	Hampshire	12 (2)	–
	Surrey	–	4 (1)
	Berkshire	–	4 (1)
South West England	City of Bristol	6 (1)	–
	Gloucestershire	–	4 (1)
	Dorset	–	4 (1)
East of England	Cambridgeshire	12 (2)	
	Hertfordshire	–	4 (1)
	Suffolk	–	4 (1)
East Midlands	Nottinghamshire	–	4 (1)
West Midlands	West Midlands	12 (2)	8 (2)
	Staffordshire	6 (1)	–
	Herefordshire	–	4 (1)
North West England	Lancashire	12 (2)	4 (1)
	Merseyside	6 (1)	8 (2)
	Cheshire	–	4 (1)
North East England	Tyne and Wear	–	4 (1)
**Scotland**			
Highlands	Inverness	–	4 (1)
Central Scotland	City of Glasgow	6 (1)	–
	Lanarkshire	–	8 (2)
**Wales**			
North Wales	Gwynedd	–	4 (1)
South Wales	West Glamorgan	6 (1)	–
**Northern Ireland**	City of Belfast	–	4 (1)

**Figure 1:**
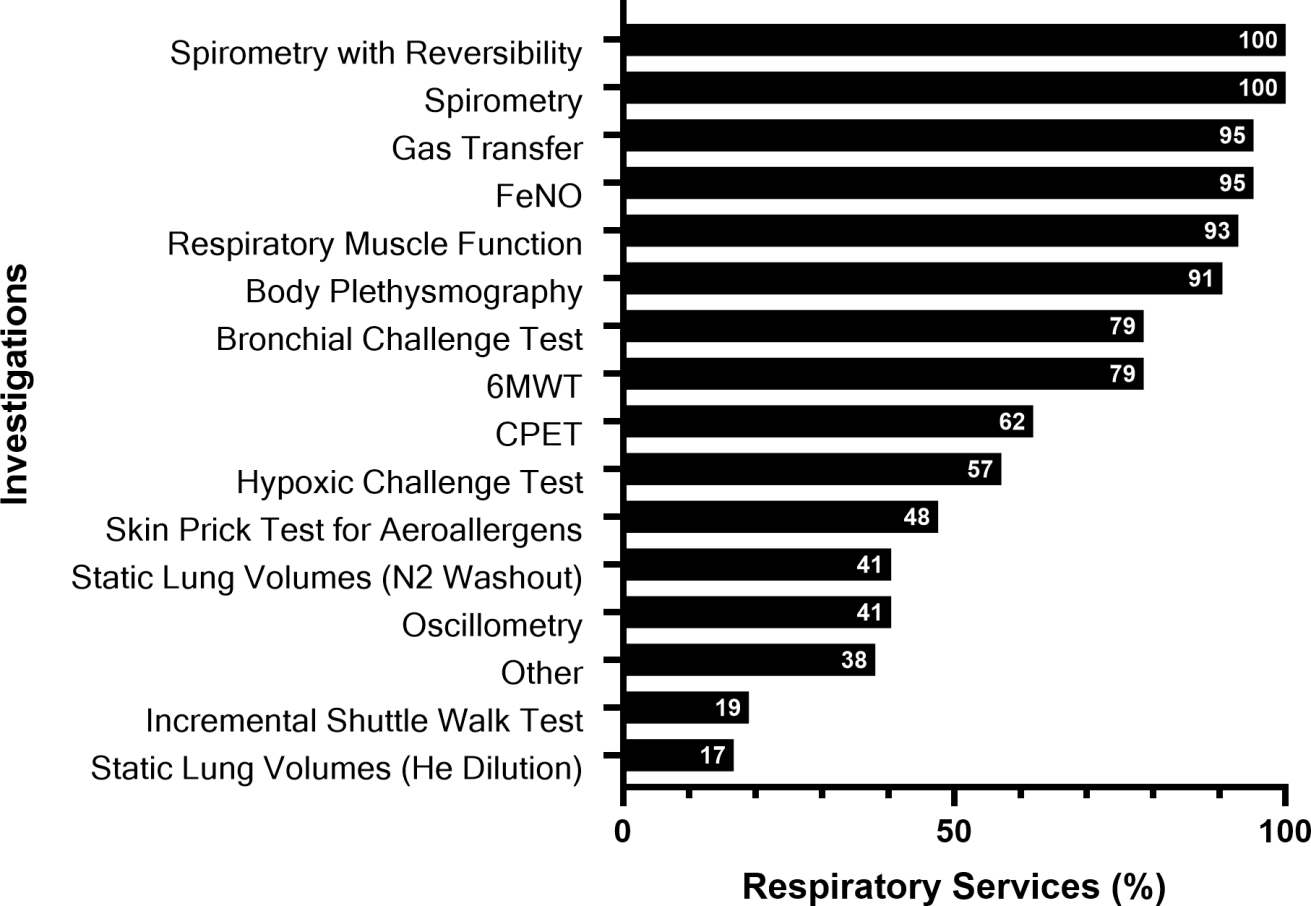
Percentage of respiratory services performing given lung function tests. 6MWT, 6-min walk test; CPET, cardio-pulmonary exercise test; FeNO, fractional exhaled nitric oxide.

Figure 1 displays the respiratory physiology tests including routine lung function tests used by responding services. ‘Other’ primarily included multiple breath washout, exercise-induced asthma or bronchoconstriction testing, incremental shuttle walk test, oxygen assessments, arterial and capillary blood gas sampling and antibiotic challenge testing.

### Current use and availability of oscillometry

Of the 42 services, 40% reported owning an oscillometry device, 57% did not, and one was unsure. Table 1 displays the geographical location of services with and without an oscillometry device. Among services with a device, 53% were reportedly based in secondary care and 47% in tertiary or specialist centres. Median full-time respiratory physiology staffing was 9 (range: 1–23) and 4 (range: 1–21) for services with and without a device, respectively.

Among device owners (n=17), 47% used a FOT (Resmon Pro), 47% used an IOS (Vyntus IOS) and one service used both. One service also reported additional use of a Tremoflo (Thorasys) device. Devices had been owned for a mean duration of 3.5 years (range: 1–7 years). Of the 94% (n=16) actively using their devices, 69% reported using it for clinical testing, 6% for research, 19% for both and 6% for ‘other’. The mean duration of device ownership was 3.2 years (range 1–7) for services using oscillometry for clinical testing only (n=11); 5.8 years (range 5–7) for those using it for both clinical testing and research (n=3); and 2.2 years (range 2–2.5) for services using it for research only, other purposes or not at all (n=3).

### Clinical indications and patient groups

Among services using oscillometry in practice (n=14), 57% use it in adults, 21% in paediatrics and 21% in both. All (100%) use it for patients with asthma. Other indications include COPD (57%), bronchiectasis (43%), cystic fibrosis (29%), interstitial lung disease (ILD; 21%), lung cancer (14%) and other conditions (29%) such as preschool wheeze and upper airway obstruction. The most common reason for use was inability to perform technically acceptable spirometry (86%). Other uses included reversibility testing (21%), bronchial challenge (7%) or as part of asthma clinic workups.

### Technical procedures and testing practice

Oscillometry was primarily performed by respiratory physiologists (88%), including trainees, clinical scientists (88%), and senior physiologists (82%). One site involved a respiratory nurse. Staff qualifications varied but most held ARTP Part 1 (77%), ARTP Part 2 (71%), STP (82%) or ARTP Practitioner accreditation (77%). Additionally, PTP (47%), ARTP Associate (47%), ARTP Spirometry (41%) and ‘other’ (18%), including STP and PTP equivalence, were held.

Among services currently using oscillometry (n=16), all (100%) reported adherence to ERS guidance^12^ for repeatability, obtaining a minimum of three trials with a coefficient of variation (CoV) ≤10% in adults and ≤15% in paediatrics for R_rs_ at the lowest oscillation frequency. While 77% of services reported always performing oscillometry prior to spirometry, the remaining services did so except when spirometry was not possible or when oscillometry was the only test being performed. Additionally, 35% reported regularly using a biological control subject as recommended in the ERS guidance, but with varied frequencies of at least monthly, to weekly and to every testing session.

Most services reported performing oscillometry using patient’s own hands for cheek support (71%), while others used operator (physiologist or technician) hands (24%) or parent hands in paediatrics (13%). Additionally, 65% did not perform slow vital capacity (SVC) manoeuvres with oscillometry, noting they were included in spirometry sessions.

### Reporting and interpretation

**Figure 2:**
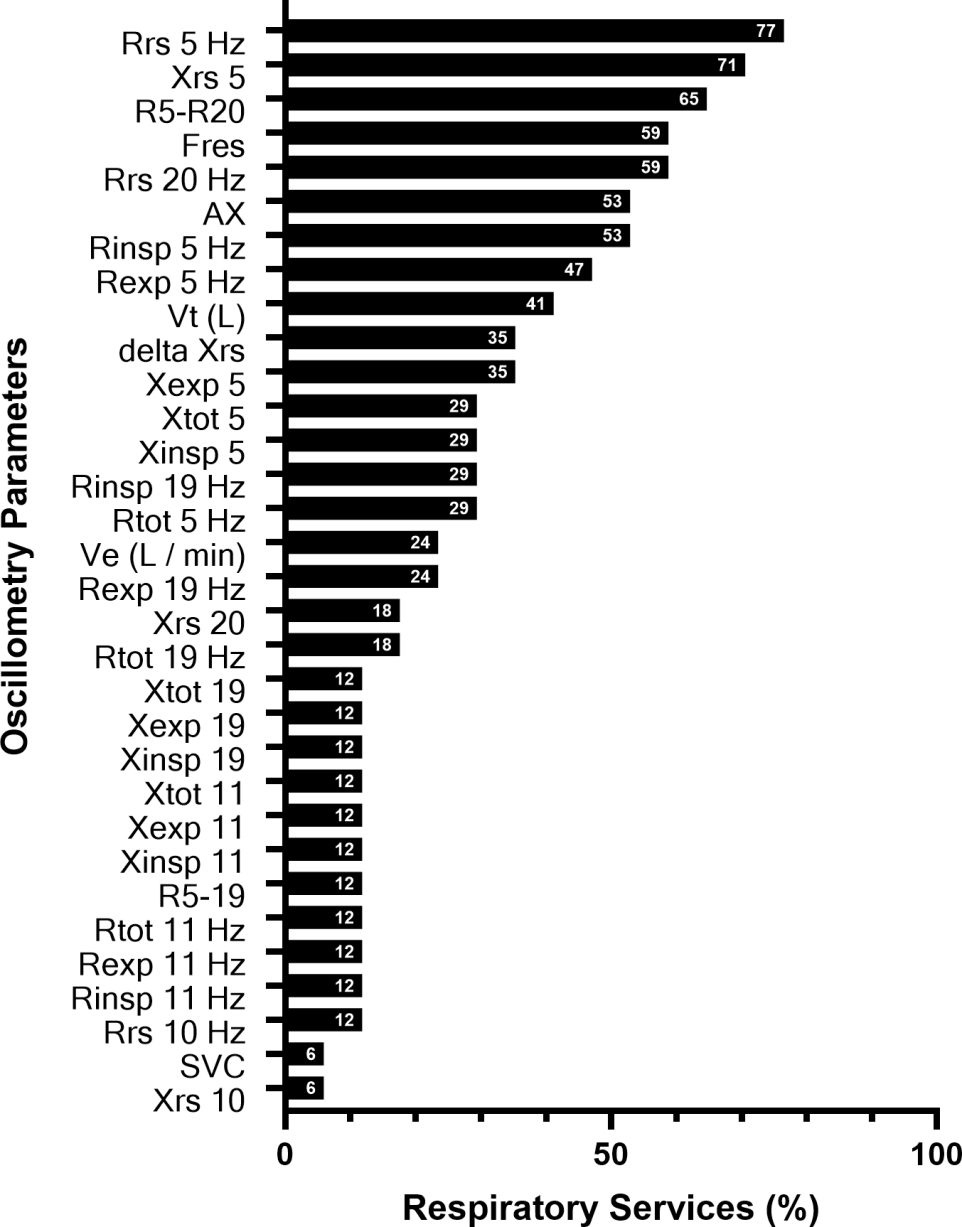
Primary reported oscillometry measurements included in a report. AX, area of reactance; exp, expiration; Fres, resonance frequency; insp, inspiration; Rrs, resistance; tot, total; Ve, minute ventilation; Vt, tidal volume; Xrs, reactance.

There was variability in how oscillometry results were reported, with 47% including technical details (device type, number of trials and the Z filter) and 35% including the make/model of device; 59% including CoV; 0% including cheek support; and 39% did not include any of these in their reporting. ‘Other’ included the generated ResMon report. Figure 2 shows the oscillometry measurements reportedly included in a report.

The most frequently reported clinical parameters were R_rs_ 5 Hz (71%), X_rs_ 5 (59%), R_rs_ 20 Hz (53%), R5-20 (47%) and AX (41%). Z-scores were used by 94% of services using oscillometry (53% for FOT, 47% for IOS), and 56% also used % predicted (44% for FOT and 56% for IOS). However, 31% of services were unsure which reference values were applied, with a further 19% reporting either the standard Vyaire or ResMon option. The primary named source was Oostveen *et al*.^25^

### Barriers to wider adoption

**Figure 3:**
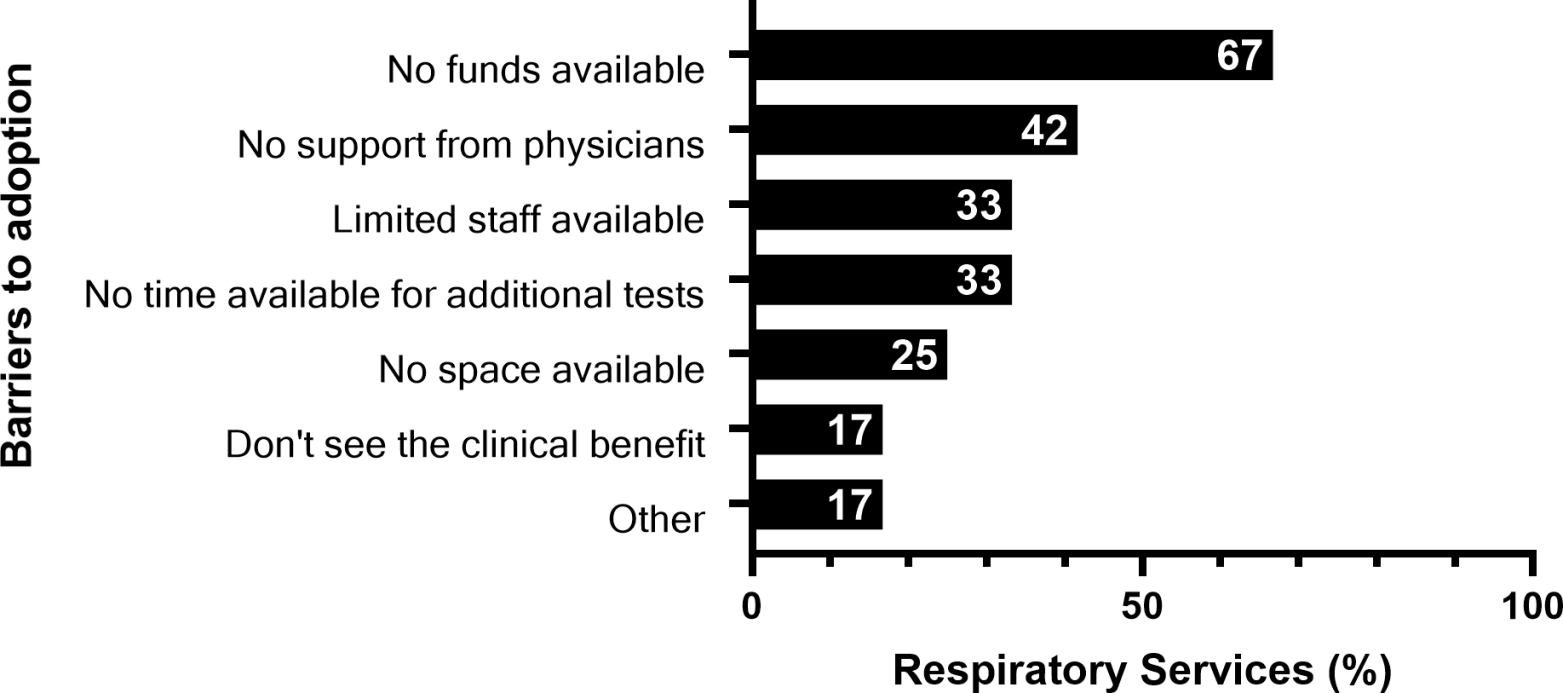
Barriers to adoption of oscillometry within clinical practice.

Among the 24 services without a device, 50% were considering a purchase within the next 5 years. The most frequently cited barriers among those not considering adoption (figure 3) were lack of funding and insufficient clinical demand.

### Training and consultant knowledge

**Figure 4:**
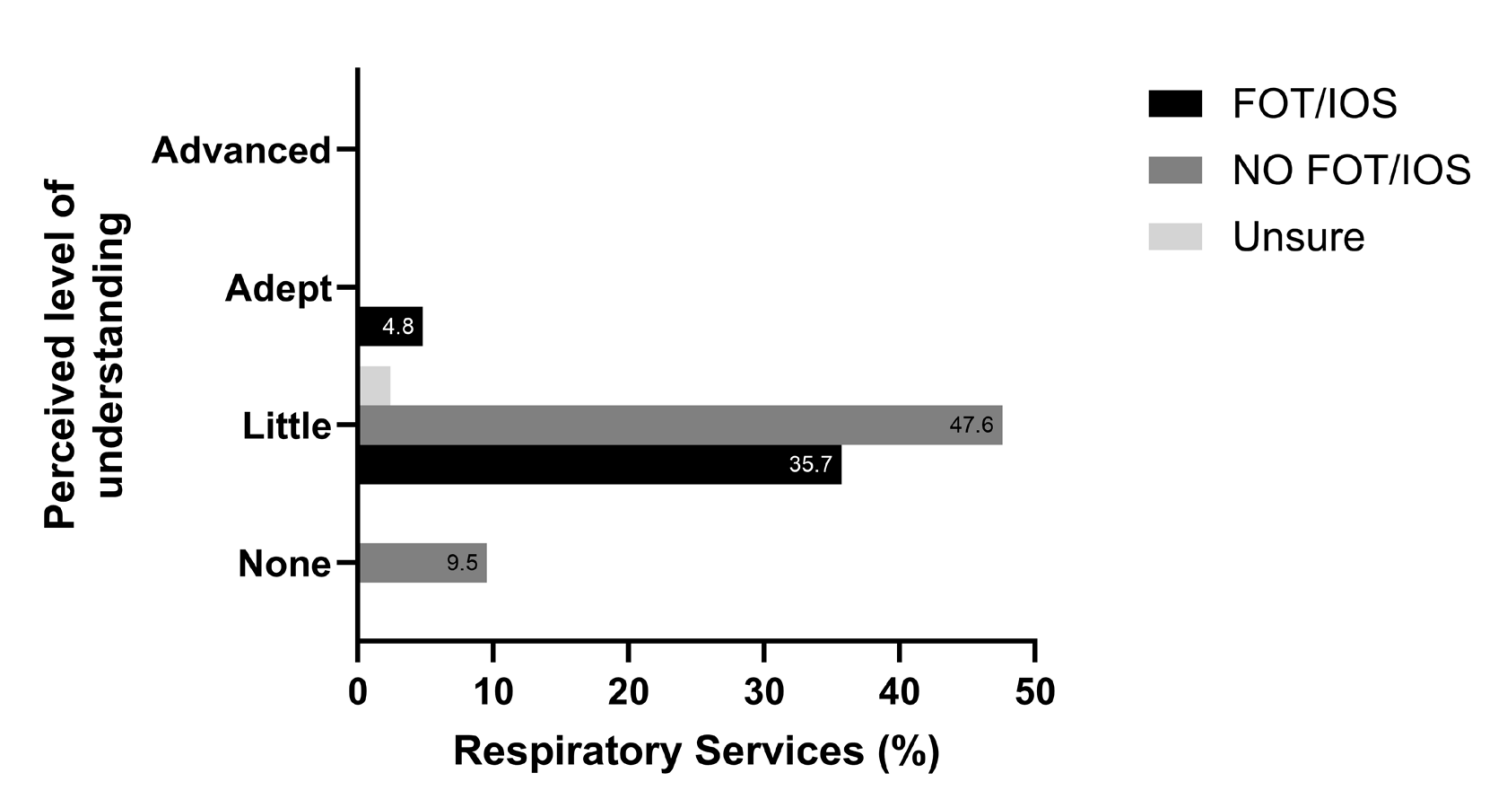
Perceived level of respiratory consultants' understanding of oscillometry for services with and without a forced oscillation technique or impulse oscillometry device. FOT, forced oscillation technique; IOS, impulse oscillometry system.

Perceived clinical understanding of oscillometry was limited, with 10% reporting that they perceived respiratory consultants had no understanding, 86% perceiving ‘little’ understanding and only 5% considered them adept. Most (74%) said consultant understanding varied within the same department, and 26% reported this to be consistent from one consultant to another. Figure 4 displays this for those with and without FOT/IOS.

Themes from free-text responses highlighted gaps in training, awareness and clinical interest (online supplemental material 3). Only 48% of respondents reported formal oscillometry training from a manufacturer. Importantly**,** 95% of respondents supported the development of ARTP guidance on oscillometry covering interpretation, limitations and reporting standards.

## Discussion

To our knowledge, this is the first survey to explore the current use, clinical indications and barriers to the implementation of oscillometry in respiratory services in the UK. Our findings demonstrate that oscillometry is already in use in 40% of surveyed NHS services with an equal distribution of FOT and IOS devices, and the ResMon Pro and Vyntus IOS being the most used.

Most respondents and device owners were based in England, with no notable geographical differences between services with and without a device, nor a clear indication of increased uptake in tertiary versus secondary care centres. A greater respiratory physiology workforce may, however, facilitate adoption. Additionally, oscillometry ownership was more common in NHS services affiliated with universities. 71% of services with a device were members of the University Hospital Association, compared with 25% of services without a device. This suggests academic affiliation may facilitate adoption, potentially through greater engagement with research, innovation and funding. Furthermore, services using oscillometry for clinical testing, alone or alongside research, reportedly owned their device longer than those using it for research only or not at all. This may suggest that greater experience supports clinical implementation. However, the strength of any causal reasoning is limited by the cross-sectional and descriptive nature of this study. Nonetheless, the adoption of oscillometry remains inconsistent, with notable variability in practice, reporting and perceived value.

### Clinical use and indications

Notably, all services using oscillometry reported doing so for asthma, indicating a strong focus on asthma within current clinical practice. Oscillometry is particularly valuable for identifying small airway disease and thus patients with poor asthma control, independent of airflow obstruction on spirometry.^5 14 16^ With just over half of surveyed NHS services with an oscillometry device (57%) also reporting use in COPD patients, and far fewer for other conditions such as ILD, this suggests a growing role of oscillometry in the assessment of airway disease across the lifespan. Given the significant burden and heterogeneity of COPD,^1 22 26 27^ expanding the use of oscillometry in routine clinical practice further could improve the understanding, early detection and phenotyping.^28^ In particular, identifying the presence of expiratory flow limitation using oscillometry^29^ is indicative of small airway disease and is associated with gas trapping and increased symptom burden.^15 30^

The most common indication for testing, aside from asthma, was in patients unable to perform technically acceptable spirometry. While this aligns with international literature supporting use in patients unable to perform spirometry, including hospitalised patients,^10 20 31^ evidence on the standalone diagnostic value of oscillometry remains mixed.^932^,^34^ Several studies suggest inadequate sensitivity and specificity compared with spirometry in some populations, reinforcing the notion that oscillometry may be best deployed as a complementary tool, as opposed to an alternative to spirometry.^9 34^ Either oscillometry or spirometry may detect airflow obstruction, while the other test may not.^35^ However, it is important to acknowledge that spirometry and oscillometry measure different physiological aspects of lung function and exhibit fundamentally different methodologies. Given its feasibility and early-detection potential in small airways disease, especially in individuals presenting with respiratory symptoms but preserved FEV₁,^21 32^ its clinical utility in targeted contexts warrants further research. Further prospective clinical trials, incorporating sensitivity and specificity analyses, are needed to improve confidence in the use of oscillometry in the absence of spirometry, particularly within the UK population.

### Technical practice and variation

While all services directly indicated adherence to the 2020 ERS Technical Standards^12^ for achieving acceptability and repeatability criteria, variability in practice remains. The ERS document published in 2020 is also due to be updated, presenting an opportunity to address inconsistencies. Over half of the services using oscillometry did not report regularly performing a biological quality control (BioQC), as recommended. However, guidance suggests weekly use of a BioQC subject for oscillometry equipment used on a regular basis,^12^ and thus this may indicate a relatively low frequency of oscillometry use in clinical practice.

Moreover, cheek support techniques varied across services, although most performed oscillometry using patient’s own hands. While recent studies suggest self-supported cheek techniques may be acceptable in healthy individuals to reduce upper airway artefacts, this may skew the results to appear worse in those with respiratory disease, such that resistance and reactance at 5 Hz have been shown to be significantly higher and significantly lower, respectively, when compared with operator cheek support.^18 21 36^ This has implications for test accuracy and reinforces the need for standardised protocols, including clearer reporting of procedural details like method of cheek support.

Most services do not perform SVC manoeuvres during oscillometry. The reasons given were that SVCs are not reportedly performed for paediatrics and are typically performed as part of spirometry testing in adults. While the performance of SVCs during oscillometry can provide a surrogate measure for closing volume, otherwise measured using single-breath nitrogen washout,^37^ its role in clinical practice remains unclear, and further investigation is needed to determine if its inclusion provides clinically valuable information.

### Interpretation

The most reported oscillometry parameters in clinical practice included resistance (R_rs_) at 5 Hz, reactance (X_rs_) at 5 Hz, area of reactance (AX) and, for IOS devices, R20 and R5-R20. Recent expert guidance from clinicians participating in an international Delphi study provided consensus for routinely reporting R5, X5 and AX in asthma and COPD, indicating these few parameters should be prioritised.^38^ They also concluded that oscillometry should be interpreted based on Z-scores when determining an abnormality for resistance and reactance at 5 Hz and AX, given percentage predicted does not account for other lung function determinants including age, sex and height. This is in support of the current survey findings where most responding services using oscillometry report on z-scores (SR values). Similarly to the Delphi study,^38^ the most used reference equation reported in this survey was that established from Oostveen *et al*.^25^ Despite the recent report on interpretation of oscillometry, there is still a clear need for more comprehensive guidance on the interpretation of results.

### Barriers to adoption and need for education

Among non-users, 50% would consider purchasing a device within the next 5 years, with cost and lack of clinical demand as the main barriers. Respiratory consultant knowledge of oscillometry was generally perceived as low, which is perhaps unsurprising given the British Thoracic Society (BTS) does not currently publish guidance or offer training on oscillometry, nor is it included in the National Institute for Health and Care Excellence (NICE) guidelines for the diagnosis and management of asthma and COPD. It is essential that healthcare professionals performing or interpreting oscillometry have a thorough understanding of the technique. As such, future guidelines and recommendations should focus on education and training, an area also previously highlighted by Sarker *et al*.^39^ Notably, nearly all respondents agreed that national guidance from the ARTP would be valuable to raise awareness, standardise practice and improve clinician understanding. Our study is therefore in agreement with Chung et al^38^ who suggested incorporating their findings on oscillometry interpretation into educational resources to increase uptake of oscillometry into clinical practice.

### Implications for practice and policy

While there is clear interest in oscillometry and some evidence of its clinical adoption, current practice is inconsistent and often unsupported by local expertise or training. Collaboration with key professional bodies, such as the ARTP, to develop national guidance is a logical and necessary next step. This should include standardised protocols for test delivery and quality assurance; minimum reporting requirements for clinical interpretation; education and upskilling of respiratory teams; and recommendations for business cases and service integration into NHS services. Such efforts will help bridge existing gaps in knowledge among respiratory physiologists but also across wider multi-disciplinary teams, in particular respiratory consultants and general practitioners.

Clinical scientist-led services offer greater capacity and flexibility to incorporate emerging technologies into clinical settings compared with services in previous generations. However, without more robust evidence demonstrating the effectiveness of oscillometry testing in clinical diagnosis and management of respiratory disease, acceptance from clinicians and thus its use in routine clinical practice will remain limited. Importantly, clinical research will need to go beyond the evidence base already established over decades for spirometry to justify widespread adoption.

Ongoing initiatives like the national Community Diagnostic Centre (CDC) programme, launched in the UK in 2021, could provide a timely opportunity to integrate oscillometry more widely into clinical care, especially if aligned with clear guidance, appropriate training and the three strategic shifts outlined in the recent 10-year Health Plan for England: hospital to community, analogue to digital and sickness to prevention.^40^ CDCs aim to expand both respiratory and non-respiratory diagnostic capacity, improve productivity and efficiency and increase accessibility by delivering services closer to patients’ homes. This includes the direct access service provided to general practitioners for asthma and COPD investigations and the adult breathlessness pathways. As part of this initiative, funding was allocated to cover resource costs to offer additional services across England. While there is no NHS or CDC tariff to perform oscillometry, and thus departments cannot claim for this test at present, it could potentially provide a route to support the financing of tests like oscillometry in the future.

There is also growing potential for the integration of tests like oscillometry in other emerging clinical pathways such as the lung cancer screening programme, by facilitating earlier detection of respiratory disease in high-risk populations, including individuals with a smoking history. Importantly, small airway dysfunction detected via tests such as oscillometry, especially where the presence of emphysema on low-dose CT scans is minimal to none,^41^ may provide objective evidence to support behaviour change, such as smoking cessation in high-risk individuals. This would again align with the NHS 10-year health plan for England with emphasis on preventing poor outcomes through early detection.^40^ However, as stated above, further prospective studies are needed to investigate the clinical utility of oscillometry, both in the diagnosis of respiratory disease and as a screening tool to support clinical decision making in the absence of spirometry.

If these are addressed, oscillometry has the potential to expand access to lung function testing, improve early detection of respiratory disease and provide a patient-friendly alternative or adjunct to spirometry. This is particularly relevant in under-served or vulnerable populations and within community settings, again in line with the 10-year Health Plan for England.^37^

### Strengths and limitations

A key strength of this study is its national scope and inclusion of qualitative insights, providing a richer understanding of clinical attitudes, barriers, and variations in practice. Moreover, it emphasises the involvement of key stakeholders, such as ARTP, in both the distribution of the survey and the dissemination of findings to the respiratory physiology community. However, despite efforts to reach participants beyond the ARTP membership, the survey invitation may have missed respiratory staff who were not ARTP members. With 42 responses, the sample represents only a proportion of NHS respiratory physiology services. Ideally, this would be expressed as a percentage of the total number of such services in the UK. However, due to the limited data available, we are unable to accurately determine the proportion of UK services represented in this study. This highlights a gap in national data that could be addressed by ARTP. It also seems likely that services without an oscillometry device were less inclined to respond. This therefore introduces potential response bias, limiting generalisability. Although up to 40% of services appear to own an oscillometry device based on the responses provided, the true proportion is likely to be much lower.

As this study focused exclusively on NHS respiratory physiology services, the findings may have limited generalisability to other healthcare settings where resources, practices and policy differ. Nonetheless, they offer valuable insights into real-world implementation of oscillometry within the UK’s healthcare system.

Although outside the scope of this study, future research could explore the number of patients undergoing oscillometry testing in UK clinical services and identify individuals responsible for interpreting the results. Future work should explore service-user and clinician perspectives and aim to validate findings in a larger cohort. Where feasible, direct engagement with all UK services may improve response rates and thus the generalisability of results.

## Conclusion

Oscillometry is currently used in some UK respiratory services, primarily for asthma and in individuals unable to perform technically acceptable spirometry. There is also some evidence of its use in COPD within clinical practice, with growing recognition of its role in small airway disease and earlier detection. Despite its potential, practice remains inconsistent, with variability in test procedures, interpretation and confidence among healthcare professionals. While these findings are limited to NHS respiratory services within the UK, they strongly support the development of national ARTP-endorsed guidance to improve awareness, standardise practice and facilitate broader clinical adoption, ultimately improving patient access to lung function testing and the early detection and management of respiratory disease.

## Supplementary material

10.1136/bmjresp-2025-003786 online supplemental file 1

10.1136/bmjresp-2025-003786 online supplemental file 2

10.1136/bmjresp-2025-003786 online supplemental file 3

## Data Availability

Data are available upon reasonable request.
