# Supplementary figures and images for: Current use and future potential of oscillometry in UK lung function testing: a national survey
Source: BMJ Open Respir Res. 2026 Jun 25;13(1):e003786. doi: 10.1136/bmjresp-2025-003786 (PMC13311712; doi:10.1136/bmjresp-2025-003786)

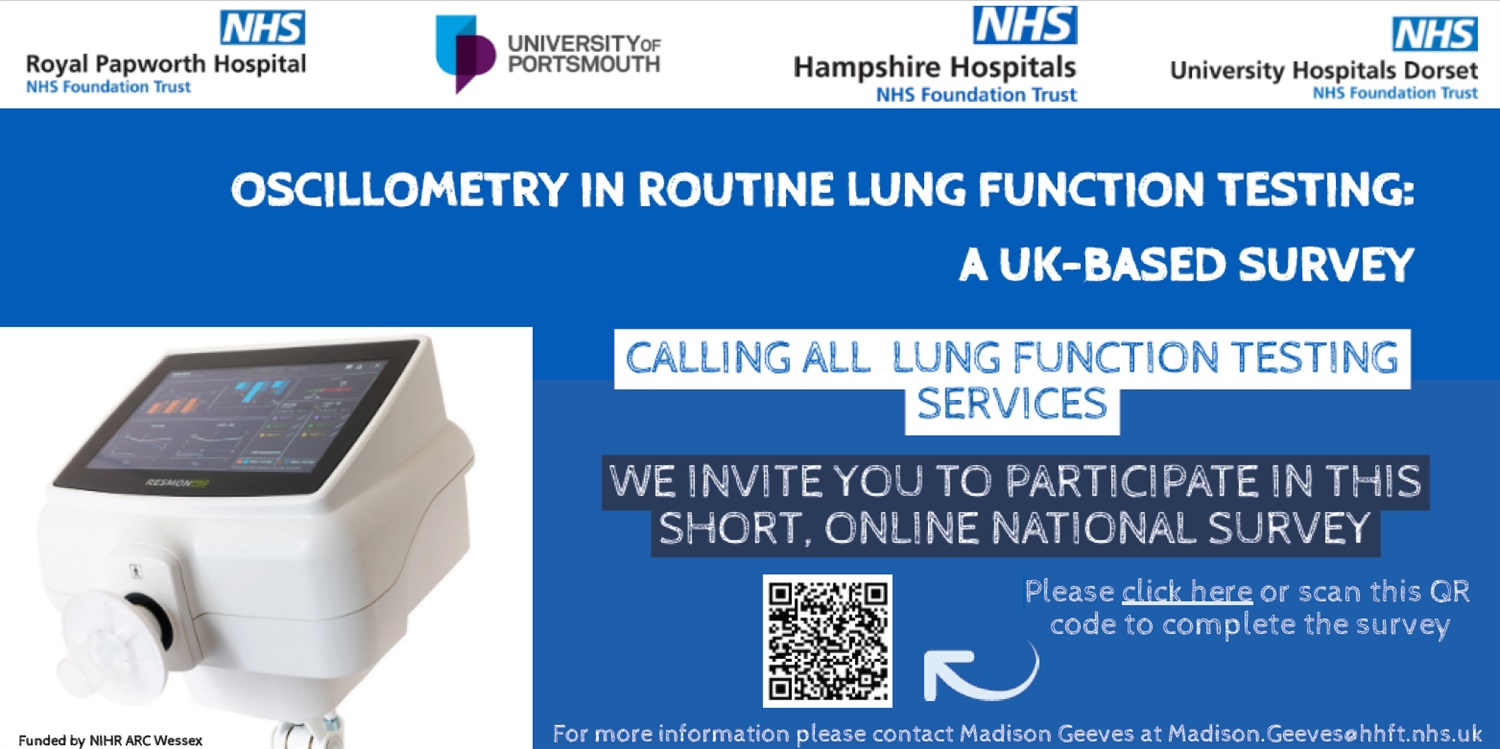

Supplement: online supplemental file 1 [file bmjresp-13-1-s001.png]
