# Supplementary material for: Current use and future potential of oscillometry in UK lung function testing: a national survey
Source: BMJ Open Respir Res. 2026 Jun 25;13(1):e003786. doi: 10.1136/bmjresp-2025-003786 (PMC13311712; doi:10.1136/bmjresp-2025-003786)
Supplement: online supplemental file 2 [file bmjresp-13-1-s002.docx]

**Supplemental Material 2: Study Information, Consent and Survey Questions**

**Study Information and Consent**

**NO CONSENT**

**CONSENT**

**Questions 1 - 4**

**Your Service**

**Questions 5**

**YES**

**NO**

**Question 6**

**Question 7**

**Question 8 - 9**

**Oscillometry**

**NO**

**Question 11**

**Question 10**

**NO**

**YES**

**Question 12 - 15**

**Technical and Interpretation Considerations**

**Question 23 - 27**

**Question 16 - 22**

**Question 28 - 30**

**Education and Training**

**End of Survey**

**End of Participation**

**Study Information**

# Study title: Oscillometry in routine lung function testing: A UK-based survey.

**Participant Information Sheet** (Version 1 06/11/2023), reviewed by the University of Portsmouth, Faculty of Science and Health Ethics committee.

**Brief Introduction:**

We would like to invite you to take part in this research study about routine lung function testing and oscillometry (also known as forced oscillation technique [FOT] or impulse oscillometry [IOS]). This is a non-invasive method that uses small-amplitude pressure oscillations superimposed on normal breathing to measure respiratory mechanics.

**Why are we doing this study and what is involved?**

Various tests are available for routine lung function testing to diagnose and monitor respiratory conditions (e.g. COPD, asthma). Spirometry has long been the gold standard for investigating and monitoring airflow obstruction. However, it requires a significant degree of coordination and maximal effort from the participant, which can be difficult in certain people – such as those who are elderly, very young or cognitively impaired.

Oscillometry measurements are made during normal tidal breathing, which may be more preferable to patients than spirometry. Furthermore, it offers additional valuable and/or alternative information about a person’s lung disease (e.g. small airways dysfunction and early disease).

We are interested in understanding the current use of oscillometry within clinical practice in the UK. We are asking staff currently working within a respiratory physiology/lung function service to complete a national survey to investigate where oscillometry is currently offered and to capture specific information on its use and performance (e.g. who, why, when and how). We anticipate the survey to take approximately 10 minutes to complete.

**Do I have to take part?**

Your participation is voluntary and the responses provided will be anonymous (albeit you will be asked to list your place of work for us to create an anonymous map of responses). You can choose to withdraw from the study at any time before submission of the questionnaire. However, please note that after submission, consent cannot be withdrawn.

**Who can take part in this study?**

- Currently working within a Respiratory Physiology (or Lung Function) Department and responsible for or participating in lung function testing (e.g. trainee or registered respiratory physiologists, practitioner training programme (PTP) or scientist training programme (STP) students, clinical scientists, research practitioner/nurse).
- **Only 1 response from each NHS trust is required (or each respiratory department if there are independent departments within the same trust). Note: this response can be completed as a team.**
- Fluent in English
- Can independently read and comprehend the survey and has digital literacy skills to complete an online survey.

**What are the possible benefits of taking part in this study?**

There are no direct benefits to you for completing the questionnaire. However, we hope the results will increase our understanding of the current use of FOT, including some of the barriers and facilitators that obstruct or promote its use and this may indirectly improve patient care in the near future.

**What are the possible disadvantages and risks of taking part?**

We do not anticipate any risks of taking part. The main disadvantage is the time you will spend completing the survey. Although no personal information is collected and thus remains anonymous, we do ask you to provide your place of work (e.g. NHS Trust and where applicable hospital site if more than one). However, these will not be named in data presented in a publication/abstract etc.

**What will happen to my results of this study?**

We will aim to publish the findings in research journals and to present them at conferences in the UK or abroad. Your data will always remain anonymous and your name will not appear on any results. All data will be stored on a password protected file.

**Contacts for further information:**

If you would like more information or if you have any queries please do not hesitate to contact us - Madison Geeves, Senior Respiratory Physiologist ([Madison.Geeves@hhft.nhs.uk](mailto:Madison.Geeves@hhft.nhs.uk)) or Dr Zoe Saynor ([zoe.saynor@port.ac.uk](mailto:zoe.saynor@port.ac.uk)).

**Consent**

- I confirm that I meet the stated participation criteria
- I understand that my participation is voluntary and that I am free to withdraw at any time prior to clicking ‘questionnaire completed’, without giving a reason
- I understand that any information given by me may be used in future reports, articles or presentations by the research team
- I understand that my name will not appear in any reports, articles or presentations
- I confirm that I have read and understand the information sheet version 1 dated 06/11/2023 for the above study and I voluntarily agree to participate in this research study.
- Please check this box to continue.

**Survey Questions**

**Section 1: Your Service**

1. a) Which NHS Trust are you employed by ……………..

b) Which hospital/department are you responding for (only applicable if there is more than one Respiratory Physiology service within your trust) ……………………..

1. What type of healthcare is your centre part of?

- Primary care
- Secondary care
- Tertiary (Specialist) care

1. Is your centre predominately adults, paediatric or mixed?

- Adult
- Paediatric
- Mixed (Adults and Paediatric)

1. How many WTE respiratory physiology workforce employees do you have within your department?

………………………………

Please provide any additional information that may be useful here (optional)

…………………………………………………

1. Which lung function tests are used in your respiratory physiology/lung function centre? (Please select all that apply)

- FeNO
- Spirometry
- Spirometry with reversibility
- Gas transfer
- Static lung volumes (body plethysmography)
- Static lung volumes (helium dilution)
- Static lung volumes (body nitrogen washout)
- Six-minute walk test
- Incremental shuttle walk test
- Skin prick test for aeroallergen
- Cardio-pulmonary exercise test
- Respiratory muscle function (any of: MIPS/MEPS/SNIP/seated vs supine VC)
- Hypoxic challenge test
- Bronchial challenge test (e.g. Methacholine, Mannitol)
- Oscillometry
- Other (please specify) ………………….

**Section 2: Oscilometry**

1. Does your department own an impulse oscillometry (IOS) and/or forced oscillation technique (FOT) device?

- Yes
  - FOT
  - IOS
  - Both
- No

Please provide any additional information here (optional) ………………………

If no to Q6 please answer Q7 and then continue to Q28

1. Is this something you would consider purchasing in the next 5 years?

- Yes
- No

If no, please provide your reason:

- Don’t see the clinical benefit
- No support from physicians
- No funds available
- No time available for additional tests
- No space available
- Limited staff available
- Other (please specify) ………………………………

Please provide any additional information here (optional) ………………………

If yes to Q6, please continue to Q8

1. Please state the device
2. Manufacturer (e.g. Vyaire, ResTech etc) ………………………………………..
3. Model (e.g. Vyntus IOS, Resmon Pro, ResMon Pro Full V3 etc) ………………………………………..
4. How long roughly have you owned this device for?

…….. years and ………… months

1. Does your centre currently use this device?

- Yes
- No

Please provide any additional information here (optional) ………………………

If yes to Q10 please continue to Q12

If no to Q10, please expand on why do you not use it and then continue to Q11…………………..

1. Has your centre previously used this device?

- Yes
- No

If no to Q11, please expand on why you have not used and then continue to Q28 ………………………………………..

1. Where is this device used within your service?

- Clinical testing
- Research
- Other (please specify) ……………………..

Please provide additional information that might be useful here (optional) ………………………………………………………..

1. What population group is this device primarily performed on within your service?

- Adult
- Paediatrics
- Both

Please provide additional information that might be useful here (optional) ………………………………………………………..

1. What conditions is it used for? Please select all that apply.

- COPD
- Asthma
- ILD
- Lung Cancer
- Cystic Fibrosis
- Other (please specify) …………………

Please provide additional information that might be useful here (optional) ………………………………………………………..

1. What is the indication of testing when used in clinical practice? Please select all that apply.

- All patients who perform spirometry
- Only if spirometry is normal
- For reversibility testing
- For patients who cannot perform technically acceptable spirometry
- For bronchial challenge testing
- Other (please specify) …………………………

Please provide additional information that might be useful here (optional) ………………………………………………………..

**Section 3: Technical and interpretation considerations – targeting standardisation**

**Testing protocols and procedures**

1. a) Who performs oscillometry testing? Please tick all that apply.

- Respiratory Physiologist (including trainee’s)
- Clinical Scientist (including trainee’s)
- Senior Respiratory Physiologists
- Respiratory Nurses
- Other (please state) ………………………..

b) What is their experience level? Please provide a range for:

1. Banding ………………..
2. Years of experience ………………..
3. Please provide all professional qualifications successfully obtained by staff members performing oscillometry? Please tick all that apply

- PTP
- STP
- ARTP Spirometry
- ARTP Associate
- ARTP Practitioner
- ARTP Part 1
- ARTP Part 2
- Other (please state) ……………………………………..

1. Is oscillometry always performed prior to forced spirometry?

- Yes
- No

Please expand ………………………………………………

1. Do you perform a minimum of 3 trials as per the recommendations from ERS Technical Standards (2019)?

- Yes
- No

If no, please give your reason(s)…………………………

1. Do you perform a minimum of 3 trials with a within session coefficient of variability (CoV; of resistance, Rrs, at the lowest oscillation frequency) of ≤ 10% in adults or ≤ 15% in paediatrics as per the recommendations from ERS Technical Standards (2019)?

- Yes
- No

If no, please give your reason(s)…………………………

1. Do you primarily perform oscillometry where the **operators** (e.g. physiologist) hands are placed on the patients cheeks or the **patients** hands are placed on their own cheeks?

- Operator
- Patient

Please expand on the above here ……………………………..

1. Do you perform an SVC manoeuvre?

- Yes
- No

Please expand on its use within your service …………………………

1. Do you regularly use a biological control subject as recommended in the ERS Technical Standards (2019)

- Yes
- No

Please expand on the above here ………………………………

**Reporting of results**

1. What do you include in your report. Please select all that apply from the below.

- Device manufacturer
- Device make and model
- Trial number - performed
- Trial number - reported
- CoV (%)
- Z Filter
- Cheek support
- Other (please specify) …………………………….

1. What primary oscillometry measurements do you include in your report? Please select all that apply.

**Rrs (resistance)**

- - 5 Hz
    - Inspiration (Rinsp)
    - Expiration (Rexp)
    - Total (Rtot)
  - 10 Hz
  - 11 Hz
    - Inspiration (Rinsp)
    - Expiration (Rexp)
    - Total (Rtot)
  - 19 Hz
    - Inspiration (Rinsp)
    - Expiration (Rexp)
    - Total (Rtot)
  - 20 Hz
  - R5 – R19
  - R5 – R20

**Xrs (reactance)**

- - 5
    - Inspiration Xinsp)
    - Expiration (Xexp)
    - Total (Xtot)
  - 10
  - 11
    - Inspiration Xinsp)
    - Expiration (Xexp)
    - Total (Xtot)
  - 19
    - Inspiration Xinsp)
    - Expiration (Xexp)
    - Total (Xtot)
  - 20
  - **ΔXrs**
  - AX (area of reactance)
- Resonance frequency (Fres)
- **Vt (L)**
- **Ve (L / min)**
- Slow Vital Capacity (SVC; L)
- Other (please state) ……………………………………………………………..

1. If a clinical report is produced what measurements from the previous list do you discuss?

……………………………………………………

1. What reference values do you refer to?

- Z-score
- % predicted
- Other (please specify)

1. What published reference values do you use? ……………………………………………….. (please state unsure if you do not know)

**Section 4: Training and Education**

1. Have you received training on oscillometry?

- Yes
- No

If yes, please expand on your training (i.e. in house, manufacturers etc)

……………………………………………………………………………………………………………………

1. What are your perceptions of respiratory consultants understanding of oscillometry in your centre?
2. overall:

- None
- Little
- Adept
- Advanced

1. between consultants:

- Consistent
- Varied

Please provide additional information here (optional) ………………………

1. Do you think that a publication/statement around FOT/IOS from ARTP and/or BTS discussing its benefits/limitations and/or reporting advice would be beneficial?

- Yes
- No

Please provide any additional comments and/or information here (optional) ………………………………………………………………………………………………………………………………………………

Thank you for taking the time to complete this survey.
