# Supplementary material for: Current use and future potential of oscillometry in UK lung function testing: a national survey
Source: BMJ Open Respir Res. 2026 Jun 25;13(1):e003786. doi: 10.1136/bmjresp-2025-003786 (PMC13311712; doi:10.1136/bmjresp-2025-003786)
Supplement: online supplemental file 3 [file bmjresp-13-1-s003.docx]

**Supplemental Material 3: Qualitative responses for questions 13-15.**

*Perceptions of respiratory consultants understanding of oscillometry in your service*

- The consultants in our asthma and general respiratory clinics are keen for the test to be introduced and supporting the bid.
- Some consultants very knowledgeable, others less interested
- 1 has a rudimentary knowledge the rest no knowledge at all
- We are in the process of giving a presentation to our consultants to increase awareness of the test and their understanding.
- We have two consultants with a particular interest - one Resp one Cardiac
- Some consultants have no idea this procedure exists.
- Some consultants are very familiar and knowledgeable on IOS, some have never heard of it and do not know how to interpret it. We have been running teaching sessions for them, and have adjusted the reports to make them easier to understand
- The same could be said of all physiology testing - we have a mix of consultants some very much into physiology some less so I'm unsure as to the level of understanding between consultants, but I would imagine it's varied as this isn't a test that we offer.
- Not sure many of our consultants understand the principles of oscillometry.
- Most consultants have limited knowledge about how ALL respiratory measurements are obtained, not just those which are less well used.
- Depends on training.
- It’s never been mentioned by consultants
- New device, few doctors able to understand the result and the complicated report, they rely on our interpretation.
- Sone consultants have a good grasp some are weak and are scared of requesting FOT as they don't understand how to interpret. We have run education sessions on this.
- One consultant during training.
- Dependent on interest.
- Some consultants were able to attend the recent demo, others weren't.
- I have never been approached by a respiratory consultant with regard to oscillometry
- Only person that can interpret is the asthma specialist
- Some consultants have special interest in IOS
- Minimal understanding
- Only those specifically interested are aware of it
- I do not believe the consultant understand FOT - some may have a broad/simple appreciation and others none
- It’s never come up for discussion, so I am unaware of their knowledge on this
- Information regarding diagnostics tends to be fixed once they reach consultant regrade, so if opinion at that time is that IOS adds little benefit, then unless there are significant advantages then this can be challenging to change.
- One consultant wrote their MD on the use of oscillometry. Others do not understand it as well.

*Whether a publication/statement around FOT/IOS from ARTP discussing its benefits/limitations and/or reporting advice would be beneficial*

- I think it would help support services trying to introduce the test, evidence that this isn’t a specialist/research test
- Would help guide clinical use
- Useful for any testing / service development intervention - should also focus not only on the technical benefits but structure a business plan around where it will costs and save money / time / resource
- useful to pass to medic staff
- I think it would be useful to have a more user friend publication. The ERS statement is really useful but sometimes difficult to understand
- To encourage greater understanding and referral
- They could then push for a business case for funding.
- It would help work towards standardising a test that is not routinely
- used within a clinical setting (has been used in research for much longer)
- WOULD ENHANCE MY UNDERSTANDING AND COULD SHARE WITH PHYSICIAN COLLEAGUES
- An official publication would encourage and educate our consultants to perhaps ask for it any standardisation of reporting is only a good thing especially with 'new' tests
- This may encourage funding to be found to purchase a device and training to be done.
- I have had brief training on it but not sure I see the need for it, any information about the benefits would be useful.
- Support from the ARTP for the introduction of alternative measuring techniques would offer validity to our case to expand services.
- Would be a useful document for testing departments as well as for consultant education.
- We can then discuss in consultants meeting
- ARTP inform a lot of what we do
- As a governing body, ARTP should release a statement of normal values, and also on the reporting
- To help promote the benefits of using FOT
- We lack clear guidance and clarification on result interpretation
- Yes some ARTP information would be helpful in understanding tests
- Would be good to have a section in the ARTP handbook
- An ARTP training course would also be beneficial
- Standardised between departments.
- Interpretation guidance.
- Pitched correctly this could be very useful but it needs to be demystified and simplified for better understanding
- Yes it would help start a conversation and provide an external reference.
- Something formal to explain would be useful.
- Unfortunately clinicians drive resp diagnostics
- It is difficult to get clinicians to adopt emerging tech.
- Only way to convince Physiologists and clinicians is to develop a reliable exemplar case and educate.
- I'm not sure it will alter how it's implemented in practice. It's been about since 50s and not ever been implemented effectively. But maybe an educational ARTP statement would add to services with current availability of oscillometry
- Yes if it was published in the BTS or ERS journals to inform Drs about this test.
- It's a poorly understood test
- Would be helpful to consolidate recent findings and recommendations.
- Definitely. This would engage respiratory consultants
- Highly needed
- It would be useful as it would provide additional support to trainees and to teams when reporting so it can be more standardised.
- It could also inform consultants in all respiratory specialisms of the benefits
- This will help provide a better understanding for the consultants and also physiologists who have little/no experience of the test
- I think many services are unaware of possible benefits
- The more consultants understand about oscillometry, the more likely they are to request it when appropriate
- may enhance the discussion around the topic and make more professionals aware of IOS/FOT
- The ARTP holds little wait within our team. Many of their 'experts' seem to be chosen based around their locality, rather than their proven expertise.
- It would be useful to be able to put this statement to the wider respiratory team to get their thoughts and whether we should take further action.

*Final comments or additional information:*

- It's a test that we as a department are keen to use more and any way of improving its usefulness would be gratefully appreciated. Main interest/referrals would be from Asthma Consultant, for their patients whom struggle with forced efforts. Trust would maybe interested if there were a national tariff for this procedure (not sure if there is one)?
- Has been on a wish list for years but other capitol expenses have had greater priority
- We are keen for our STP students to be involved in any research and development around the expansion of our services.
- IOS appears to be a more widely utilised investigation in paediatric respiratory medicine than adult, and this needs to change.
- One can see this is a very useful test. Glad that someone it trying to make a difference!
- Oscillometry is unlikely to replace the more traditional tests such as spirometry but it can be a useful additional/alternative test, particularly for certain groups due to its non-volitional. There is also evidence to suggest it is a more sensitive marker of airway physiology than spirometry - it may be useful in early disease.
